# Investigating the Clinical Profile of Suicide Attempters Who Used a Violent Suicidal Means

**DOI:** 10.3390/jcm11237170

**Published:** 2022-12-02

**Authors:** Marlehn Lübbert, Lydia Bahlmann, Thomas Sobanski, Alexandra Schulz, Ulrich W. Kastner, Martin Walter, Fabrice Jollant, Gerd Wagner

**Affiliations:** 1 Department of Psychiatry and Psychotherapy, Jena University Hospital, Philosophenweg 3, 07743 Jena, Germany; 2 Department of Psychiatry and Psychotherapy, University of Rostock, 18051 Rostock, Germany; 3 Department of Psychiatry, Psychotherapy, and Psychosomatic Medicine, Thüringen-Kliniken, “Georgius Agricola” GmbH, Rainweg 68, 07318 Saalfeld, Germany; 4 Department of Psychiatry and Psychotherapy, Helios Fachkliniken Hildburghausen, Eisfelder Str. 41, 98646 Hildburghausen, Germany; 5 Department of Psychiatry, School of Medicine, University Paris-Saclay & Academic Hospital (CHU) Bicêtre, APHP, 94270 Le Kremlin-Bicêtre, France; 6 Moods Team, INSERM, UMR-1178, CESP, 94270 Le Kremlin-Bicêtre, France; 7 Department of Psychiatry, CHU Nîmes, 30900 Nîmes, France; 8 Department of Psychiatry, McGill University, Montreal, QC H3A 0G4, Canada

**Keywords:** suicide, suicidal attempt, violent suicide means, impulsivity, anger, aggression

## Abstract

In our study, we aimed to explore the profile of the high-risk subgroup of suicide attempters that used a violent means compared to suicide attempters that chose a non-violent suicide means. Therefore, we recruited a sample of inpatients with recent suicide attempts in three psychiatric hospitals in Thuringia, Germany. We used a structured clinical interview to assess the psychiatric diagnoses, sociodemographic data, and characteristics of the suicide attempt. Furthermore, we used several validated clinical questionnaires to measure suicidal ideations, suicide intent, depression severity, hopelessness, impulsivity, aggression, anger expression, and childhood trauma. We compared 41 individuals using violent means to 59 using non-violent means with univariate and multivariate statistical analyses. We found significantly (corrected for multiple comparisons) higher levels of impulsivity-related sensation-seeking in violent suicide attempters in univariate and multivariate analyses, and additionally in anger expression directed inward at an uncorrected statistical threshold. Besides that, there were no significant differences between the two groups. We assume that underlying neurocognitive mechanisms, such as impaired decision-making processes and/or differences in risk/loss assessment, could explain the higher levels of questionnaire-based sensation-seeking in subjects who use violent suicide means. Further research is needed, including neuroimaging and biochemical techniques, to gain more insight into the mechanisms underlying the choice of a suicidal means.

## 1. Introduction

Every 40 s a person dies from suicide, which leads to more than 700,000 suicide victims each year in the world [1]. In addition to the suffering of the suicide victims themselves, this has a devastating psychological, social, and financial impact on families and society. Despite the exponential increase in the number of publications in the field of suicidology in recent years [2], the prediction of future suicidal behavior (SB) and treatment of individuals after a suicide attempt (SA) remain challenging [3]. For instance, the suicide rate in the first 3 months after psychiatric hospital discharge is 100 times higher than the global suicide rate, particularly among patients admitted with SB, indicating a strong need for better prediction and treatment [4].

Several previous studies focused on the exploration of specific clinical and personality factors that might enhance the risk of SB. However, a recent meta-analysis markedly revealed that 50 years of research had improved our knowledge about SB and suicidal ideations (SI), but with limited impact on the prediction of future suicidal acts [5]. The only clinically robust predictor for future SB until now is a personal history of previous suicide attempts [5,6,7,8]. Scientific research, as well as clinical experience, clearly recognize that suicide attempters cannot be seen as one homogeneous group because of the significant variability in clinical factors, sociodemographic aspects, and personality profiles, as well as regarding specific characteristics of the suicide attempt [9,10]. Therefore, a better understanding of SB requires more studies investigating the heterogeneity within the suicide attempters’ group, for example, by examining subgroup profiles.

In the present study, we focused on suicide attempters who used violent means to die in order to identify specific clinical and personality variables that may characterize this subgroup. The use of a violent means seems to be an essential risk factor for death by suicide [11]. A significantly greater number of suicides in individuals using violent than non-violent methods was reported [12]. Furthermore, people who have used violent suicidal means are at increased risk of dying from suicide during the following year in comparison to those who used non-violent means [13]. Thus, suicide attempters who used violent suicidal means seem to form a specific subgroup with very high-risk levels for death in future SB and therefore might be “one step closer to suicide completers” [14]. To provide effective prevention strategies, a better understanding of the specific characteristics of such a high-risk subgroup is strongly required.

Therefore, in the present study, we aimed to investigate potential differences in demographic, clinical, and specific personality profiles between violent suicide attempters as opposed to suicide attempters who used non-violent means. Based on previous descriptions of violent suicide methods, i.e., Åspergs criteria [15], we defined violent suicide means by the use of one of the following methods: hanging, the use of firearms, jumping from heights, several deep cuts, car crash, burning, gas poisoning, drowning, electrocution, and jumping under a train. Drug overdose was considered a non-violent method [15]. Several studies are based on this selection or at least on similar definitions [14,16,17] and examine specific characteristics of these subjects. However, there are some other studies that defined “gas poisoning” to be a non-violent method of suicide [18,19]. More recently, Punzi et al. [20,21] considered that some methods, such as asphyxia, may be considered both violent and non-violent means according to the circumstances. In the present study, we followed the Åsperg criteria, but we checked whether our main results changed when we changed the group assignment considering suicide attempts by exposure to gas or vapor as a non-violent means.

Regarding sociodemographic characteristics, it was previously shown that violent compared to non-violent suicide attempters are more often male [14,18,22], older [14], living alone [23], and unemployed [24]. Furthermore, violent suicide attempters had significantly more family histories of suicide [14]. Thus, the sociodemographic factors resemble those of the suicide victims [14]. Regarding the relationship between the use of suicide means and the presence of a specific psychiatric disorder, a stronger association between violent suicide attempters and a psychotic disorder was reported, but not with anxiety or affective disorders [25]. The presence of a depressive disorder does not seem to specifically affect the choice of suicide means [26]. Despite that, circumstantial factors, such as access to specific methods, may partly explain the choice of the method [18,27]. There is also some evidence from neurobiological studies that individuals who used a violent suicide means may have a different neurobiological profile including genetic [28], biochemical [15], and brain functioning abnormalities [29].

There are no studies up to now investigating specific personality traits, i.e., impulsivity, aggression, and anger expression in violent compared to non-violent suicide attempters. We focused on these specific personality features because abnormal impulsive-aggressive traits and anger expressions have been consistently associated with suicidal behavior in previous studies [30,31]. There is also no consensus for putative associations between specific personality traits and the use of a suicide method based on concepts of lethality and seriousness. A common hypothesis is a presumed association between aggression as a trait and self- (or other-) directed violence. However, in their review, Ludwig and Dwivedi [16] did not find any conclusive evidence for the positive associations between aggressive traits and the choice of violent suicide means. In the present study, we explored putative differences in demographic, clinical, and specific personality features between violent and non-violent suicide attempters. Based on the current state of research, we expected to find significant group differences in some of the mentioned variables. Notably, we hypothesized that violent suicide attempters would be more often males, older, and living alone. Furthermore, in an exploratory analysis, we investigated the association between the use of violent suicide means and specific personality characteristics, i.e., impulsivity, aggression, anger expression, as well as hopelessness. In addition, we exploratory examined whether there were differences in the motives leading to suicide attempts between the two groups of suicide attempters.

## 2. Materials and Methods

### 2.1. Subjects

For the present study we used complete demographic, clinical, and personality questionnaires’ data from 100 adult inpatients who recently attempted suicide. It was part of a suicide prevention project (“Network for suicide prevention in Thuringia”) funded by the federal ministry of health (BMG) that was conducted from 2018 to 2020 [9]. In the absence of previous studies that examined differences in the personality traits between violent and non-violent suicide attempters, we did not have effect sizes to calculate the desired sample size based on a power analysis before conducting the study.

We contacted patients as soon as possible, on average two weeks after their suicide attempt, during their inpatient hospitalization in adult psychiatric hospital. We only included subjects who fulfilled the DSM-5 [32] criteria for a current suicidal behavior disorder (SBD). In the DSM-5, “suicide attempt” is explicitly defined as “a self-initiated sequence of behaviors by an individual who, at the time of initiation, expected that the set of actions would lead to his or her own death.” The intent to die was assessed using the Suicide Intent Scale, SIS [33]. Exclusion criteria for the present study were acute psychosis, acute intoxication, withdrawal symptoms, diagnosed intelligence impairment, language barriers, lack of insight, and dementia diseases. Table 1 depicts information about the sample characteristics. The local ethics committees of the Friedrich-Schiller University, Jena, and of State Chamber of Physicians of Thuringia, Germany approved the study. Informed written consent was obtained from all participants before their participation. Patients were contacted and interviewed by trained psychologists with a master’s degree (M.L., L.B., and A.S.). Subsequently, questionnaires were explained and given to the patients to be filled out in the following days.

### 2.2. Clinical Assessment Tools

The data collection included structured interviews and a comprehensive battery of questionnaires to measure clinical symptoms and personality traits. All psychometric scales utilized were validated and have been extensively used.

The presence of psychiatric diseases was assessed by trained psychologists using the Mini International Neuropsychiatric Interview, M.I.N.I. German Version 5.0.0 [34], a short structured diagnostic interview for DSM-IV Axis I disorders. We also collected systematic information about the sociodemographic characteristics, the number of previous suicide attempts, any familial history of SB among first-degree relatives, medication status, and the motives of the recent suicide attempt. Suicidal ideations during the past week were assessed using the German version of the Beck Scale for Suicide Ideation, BSS [35]. Depressive symptoms were evaluated by the Montgomery Åsberg Depression Rating Scale, MADRS [36], and via self-report using the Revised Beck Depression Inventory, BDI-2 [37]. Hopelessness was measured by the revised version of the validated German Beck Hopelessness Scale [38], based on Beck’s cognitive theory of depression [39], which explores pessimism with regard to the future. Impulsivity was assessed by a German version of the Impulsive Behavior Scale, UPPS [40], which explores the following four dimensions of impulsivity: lack of premeditation, urgency, sensation-seeking, and lack of perseverance. To evaluate aggressive traits and various areas of anger, a short version of the validated and widely used German Questionnaire for Assessing Factors of Aggression, K-FAF [41] and the State-Trait Anger Expression Inventory-2, STAXI-2 [42], were used, respectively. Finally, we also assessed childhood trauma using the Childhood Trauma Scale, CTQ [43].

### 2.3. Statistical Analysis

SPSS Version 26.0 (https://www.ibm.com/de-de/analytics/spss-statistics-software, accessed on 16 February 2022) was used for all statistical analyses. To investigate differences in categorical variables, i.e., in sociodemographic factors and motives, a χ^2^-test was applied. In addition, the non-parametric Mann-Whitney U-test was utilized for measures with a non-Gaussian distribution. To explore the assumed differences in clinical variables (e.g., SI, hopelessness, depression) we used a two-sided Student’s t-tests. A Bonferroni correction was applied for controlling Type I error due to multiple comparisons. We did not control for potential confounders, such as age and gender education, because we did not find any differences in these variables. We further used the discriminant analysis, as a multivariate model, to differentiate violent from non-violent suicide attempters based on the assessed clinical and personality questionnaires with continuous variables. Discriminant analysis is used when groups are known a priori and builds a predictive model for group membership to find a linear combination of variables that provide the best discrimination between the two studied groups of suicide attempters. The resulting combination can be used as a linear classifier. We included the scores of the following questionnaires with the corresponding subscales in the discriminant analysis: SIS, BSS, MADRS, BDI-II, H-R-Scale, UPPS, K-FAF, STAXI-2, and CTQ.

## 3. Results

### 3.1. Sociodemographic Data

We did not find any significant differences in the sociodemographic characteristics between the two groups of attempters (see Table 1). Around 50% of the patients in both groups were females. Patients in both groups were on average about 40 years of age, more than 50% were employed (slightly more violent suicide attempters), about 25% were married, and about 40% were living alone. In both groups, approx. 10% of the patients reported having first-degree relatives with suicidal behavior.

### 3.2. Mental Disorders

There were no significant differences between the violent and the non-violent suicide attempters regarding psychiatric disorders and the severity of depression. Most of the patients (about 65% in both groups) were diagnosed with a major depressive disorder (MDD) (see Table 1), with a similar degree of depression severity as measured by MADRS and BDI-II indicating moderate levels of depression (see Table 2).

### 3.3. Suicidal Ideation and Behavior

Subjects with a violent suicide attempt reported slightly more suicidal ideations (but not significantly more) compared with the group of non-violent suicide attempters (Table 2). There were no significant group differences regarding the number of previous suicide attempts (see Table 1). Neither the level of suicide intent nor the assessment of the lethality of the chosen method differed significantly between the two groups (Table 2).

### 3.4. Personality Traits

Regarding specific personality characteristics, we only found a significant difference between the violent and the non-violent suicide attempters in the subscale “sensation-seeking” of the UPPS impulsivity questionnaire, indicating higher levels of sensation-seeking in violent suicide attempters. There were no significant differences between the two groups regarding the level of various aggression or anger states and traits (K-FAF, STAXI-2) and other impulsivity dimensions (UPPS), except “Anger Expression-In”, which significance however did not survive correction for multiple comparisons (see Table 2). In order to test whether the different grouping would change the main result, we re-analyzed the comparison regarding “sensation seeking” with the regrouped samples. In total, four subjects attempted suicide by exposure to gas or vapor (ICD-10 codes: X66 and X67), who are now included in the group of non-violent suicide attempters. The difference between the two groups of attempters remained highly significant (*p* = 0.002).

### 3.5. Main Motives of the Current Suicide Attempt

In both groups, hopelessness, interpersonal conflicts, and other acute stressful events were reported as the most frequent motives for the current suicide attempt (see Table 1). Interpersonal conflicts were reported more often in the group of non-violent suicide attempters whereas patients using violent suicide methods reported more frequently hopelessness and acute stressful events as their main motives. However, we did not find any significant group differences regarding the main motives. Disconnectedness and perceived burdensomeness, as two factors of the interpersonal theory of suicide [44], were rarely reported as the main motives in both groups.

### 3.6. Mutivariate Analysis

Finally, as indicated by the univariate statistics, the multivariate stepwise discriminant analysis confirmed that among the clinical and personality questionnaires used, the UPPS subscale “sensation-seeking” was the only factor that significantly discriminated between the two groups (Wilks ʎ = 0.92, F (1, 89) = 8.11, *p* = 0.005). Using step-wise discriminant analysis with the regrouped samples (considering gas poisoning as a non-violent method), the variables, “sensation seeking”, and additionally “anger expression-in” were identified as the two factors that significantly discriminated between the violent and non-violent suicide attempters (in the first step with “sensation seeking” Wʎ = 0.92, F = 7.38, *p* = 0.008, in the second step with “anger expression-in” Wʎ = 0.88, F = 5.90, *p* = 0.004). The standardized canonical discriminant function coefficients were for “sensation seeking” 0.77 and for “anger expression-in” 0.62.

## 4. Discussion

The use of violent means is critical to the risk of death following a suicidal act. People who died from suicide more often used violent means, while suicide attempt survivors more often used non-violent means [11,13,45]. Even when people do not die from their violent suicidal act, they are more likely than non-violent suicide attempters to die from suicide in the following year [46]. In the present study, we aimed to identify a specific clinical profile that may characterize subjects who use violent means. The main finding was that violent in comparison to non-violent suicide attempters showed only a higher level of sensation-seeking, but were not different in other UPPS impulsivity subscales or other clinical/personality questionnaires used. In contrast to our expectations, the groups did not differ in their levels of intent to die, aggression and anger expression, rates of comorbid mental disorders and severity of depression, sociodemographic features, first-degree family history of suicidal behavior, motives, hopelessness, and characteristics of their last suicidal act (lethality, intent).

The UPPS [47] is conceptualized within the framework provided by the five-factor model of personality [48] and consists of four distinct impulsivity dimensions. The concept behind sensation-seeking incorporates the following two main aspects: a tendency to enjoy and pursue exciting activities and an openness to trying new experiences that may or may not be dangerous. Individuals with high scores enjoy taking risks and engaging in dangerous activities, whereas low scorers avoid risk and danger [47]. Previous studies showed that high scorers in sensation-seeking are more prone to drug abuse, engaging in risky sexual behaviors or excessive gambling [49,50,51], reckless driving [52], and risky sports activities [53]. Furthermore, individuals with high sensation-seeking seem to exhibit a stronger orienting response and greater cortical arousal in response to intense visual or auditory stimuli [54]. Previous studies using UPPS to investigate the association between impulsivity and suicidal behaviors produced inconsistent findings [55]. The most robust results were found for the subscale of negative urgency, showing a relationship with suicidal ideations and attempts [55]. To our knowledge, only one study has reported a significant association between sensation-seeking and the lifetime number of suicide attempts in patients with borderline personality disorder [56].

It is conceivable that the association between sensation-seeking and the use of a violent suicide means might be caused by its proxies related to the process of decision making, including risk and gain/loss evaluation, as well as specific neurobiological differences. Higher sensation-seeking scores in violent suicide attempters may represent a clinical correlate of risky decision-making, previously shown to be associated with the use of violent suicidal acts [57,58]. A recent meta-analysis outlined that violent suicide attempters may have more impairments in decision-making than subjects using non-violent suicide means (mainly medication overdose) [59]. Furthermore, a recent fMRI study found that resting-state functional connectivity, mainly between the medial orbitofrontal cortex and anterior cingulate gyrus, predicted sensation-seeking in 414 individuals. Both regions are crucially involved in processing reward-related stimuli. In both groups of suicide attempters and suicide relatives, risky decision-making was associated with deficient risk encoding in the orbitofrontal cortex [60,61] and striatum [62]. In a morphometric study, we recently observed changes in the volume of the striatum, which were more prominent in subjects using violent than non-violent suicide methods [29].

Another study using the Balloon Analogue Risk Task and electroencephalography showed that sensation-seeking moderated the effect of reward/loss magnitude on uncertainty-bearing behavior. Individuals with higher sensation-seeking scores were hyposensitive to potential losses. Some previous studies [63], although not all [64], found a reduced loss aversion in suicide attempters. Individual variation in loss aversion was associated with resting-state activity in the striatum, among other brain regions [65]. Thus, the reduced loss aversion in sensation-seekers may be particularly important in violent suicide attempters.

In sum, sensation-seekers may exhibit alterations in specific aspects of decision-making and associated brain regions. Violent suicide attempters seem to be hyposensitive to potential losses and hypersensitive to intense stimuli. Deficient dopaminergic (and maybe serotonergic) inputs to the striatum and orbitofrontal cortex may influence decision-making processes and sensation-seeking, and thus contribute to the choice of a more immediately rewarding option (e.g., in terms of rapid termination of mental pain), over subsequent losses (the end of life), when coupled with suicidal ideas and/or psychopathology.

We additionally hypothesized differences in personality traits such as aggression (in particular self-aggression) and anger expression, with higher levels in subjects using violent suicide means. However, we did not find any significant differences between the two groups regarding aggression-related traits, although it seems that individuals using violent methods had higher levels of anger directed inward. This difference could also be observed in the additional discriminant analysis with the regrouped samples, which included gas poisoning as a non-violent means. However, the significance of this result should be tested in future studies using a larger sample size. Thus, the expression of self-aggressiveness or anger does not seem to directly influence the choice of suicide means. On the other hand, we cannot interpret this finding in terms of actual aggressiveness or anger levels before or during the suicidal act, which might be unrelated to the aggressiveness/anger levels assessed by the questionnaires after the attempt. In contrast, as revealed in our previous study, self-aggressiveness plays a significant role in the repetition of suicide attempts [9].

We also expected higher levels of hopelessness in the group of violent suicide attempters. However, we found similar levels of hopelessness between the two attempters’ groups, in agreement with a very recent study [66]. Thus, the level of hopelessness does not seem to be linked to the use of the suicide method. Both groups reported hopelessness, interpersonal conflicts, and acute stressful events most frequently as the main motives for attempted suicide, with no significant differences in the violent compared to the non-violent suicide attempters. Furthermore, the violent attempters reported higher (at a trend level) levels of suicidal ideation after their current suicide attempt than subjects using non-violent means. This finding did not come with corresponding differences in the suicidal intent, as was recently reported [66], or the estimation of the lethality of the chosen method, which did not differ between the two groups.

### Limitations

Data were collected using a cross-sectional design. Thus, we cannot interpret our results in a predictive way. There might be a selection bias due to our exclusion criteria, reducing the generalizability of our results. Since study participation was voluntary, our sample was potentially biased by patients who were more open to reporting about their suicide attempt. Patients unwilling to talk about their suicide attempt might be at higher risk for future suicidal behavior, potentially showing a more negative reaction toward their survival. Furthermore, we did not find differences in most of the demographic and clinical variables as well as personality characteristics studied. Besides the true effect, this also could be related to the sampling bias. The inpatient admission numbers of subjects after a suicide attempt in a psychiatric hospital alone cannot represent the entire sample of suicide attempters. We still know little about the extent to which outpatient support systems or counseling centers instead cared for patients after a non-violent suicide attempt or whether patients received initial care in an emergency department after a suicide attempt without being referred to psychiatry for further treatment. However, it is common for almost every patient to be referred to a psychiatric hospital participating in the project “Network for suicide prevention in Thuringia” for further evaluation of suicide risk. Furthermore, violent means were defined based on Åsberg’s list. This definition of a violent suicide attempt remains controversial, perhaps leading to different results in studies based on other criteria. However, when the comparison was reanalyzed with respect to the UPPS “sensation seeking” and when the discriminant analysis was performed with the regrouped samples that included gas poisoning as a non-violent means [19], similar results were found.

Additionally, we did not investigate the underlying processes leading to the higher levels of sensation-seeking in the violent suicide attempters. Since the choice of a violent means is associated with a higher lethality, subjects who were recruited here may not be representative of all individuals who use a violent suicidal means, explaining the limited number of significant differences. Finally, this might also be due to the relatively small sample size, especially in the group of violent suicide attempters. In the absence of previous studies that examined differences in the personality traits between violent and non-violent suicide attempters, we did not have effect sizes to calculate the desired sample size based on a power analysis before conducting the present study.

## 5. Conclusions

In the present study, we found higher levels of sensation-seeking among individuals using a violent relative to non-violent suicide means. We hypothesize that underlying neurocognitive mechanisms, such as impaired decision-making processes and/or differences in risk/loss assessment, could explain the significantly higher levels of sensation-seeking in the violent suicide attempters. Further research is needed, including neuroimaging and biochemical techniques, to gain more insight into the mechanisms underlying the choice of a suicidal means.

## Figures and Tables

**Table 1:** Comparison of sociodemographic and clinical characteristics between suicide attempters who used a violent suicidal means (VSA) and those who used non-violent suicidal means (NVSA).

Characteristics	VSA (*n* = 41)	NVSA (*n* = 59)	Test Statistic	*df*	*p*
Gender (% males)	22 (53.7%)	30 (50.8%)	*χ*^2^ = 1.618	2	0.445
Age (years) (Mean ± SD)	38.93 ± 17.68	42.36 ± 17.81	*t* = −0.950	98	0.172
Number of past suicide attempts (Mean ± SD)	0.59 ± 0.50	0.46 ± 0.50	*t* = 1.254	98	0.397
Education (in years)	10.51 ± 1.44	10.45 ± 1.39	*t* = 0.219	96	0.784
Employed (%) ^a^	25 (61.0%)	31 (52.5%)	*χ*^2^ = 0.698	1	0.403
Family status					
Unmarried	23 (56.1%)	32 (54.2%)			
Divorced	3 (7.3%)	6 (10.2%)			
Widowed	1 (2.4%)	3 (5.1%)			
Married, but living apart	3 (735%)	5 (8.5%)			
Married	10 (24.4%)	13 (22.0%)			
Living alone (%)	17 (41.5%)	22 (37.3%)	*χ*^2^ = 0.398	1	0.528
1st degree relatives with suicidal behavior (%)	4 (9.8%)	6 (10.2%)	*χ*^2^ = 0.005	1	0.946
Psychiatric Disorders (main diagnosis)					
Major depressive disorder	27 (65.9%)	38 (64.4%)			
Borderline personality disorder	4 (9.8%)	4 (6.8%)			
Substance use disorder	3 (7.3%)	7 (11.9%)			
Bipolar disorders	1 (2.4%)	4 (6.8%)			
Adjustment disorder	5 (12.2%)	3 (5.1%)			
Acute stress disorder/PTSD	1 (2.4%)	0			
Phobic disorder	0	0			
Obsessive compulsive disorder	0	1 (1.7%)			
Autism spectrum disorder	0	1 (1.7%)			
Pathological gambling	0	1 (1.7%)			
Main motives for suicide attempt					
Interpersonal conflicts	8 (19.5%)	20 (32.0%)	*χ*^2^ = 2.483	1	0.115
Acute stressful events	12 (29.3%)	9 (14.0%)	*χ*^2^ = 2.864	1	0.091
Hopelessness	13 (31.7%)	15 (24.0%)	*χ*^2^ = 0.474	1	0.491
Burdensomeness	3 (7.3%)	2 (3.0%)			
Disconnectedness	0	2 (3.0%)			
Persistent stressful Circumstances and experience of overstrain	2 (4.8%)	3 (5.0%)			
Psychiatric symptoms	1 (2.4%)	4 (6.0%)			
Somatic symptoms	3 (7.3%)	0			
Fear of the future	0	2 (3.0%)			

^a^ Being employed include fulltime or part-time employment, guarded employed, volunteering work, federal volunteer service, and being in training or retraining, students and pupils. Abbreviations: SD, standard deviation; VSA, violent suicide attempters; NVSA, non-violent suicide attempters; PTSD, post-traumatic stress disorder.

**Table 2:** Comparison of clinical characteristics between suicide attempters who used a violent suicidal means and those who only used non-violent suicidal means.

	VSA (*n* = 41) M ± SD	NVSA (*n* = 59) M ± SD	Test Statistic	*df*	*p*
Montgomery-Åsberg Depression Rating Scale (MADRS)	21.17 ± 10.49	20.22 ± 10.01	*t* = 0.458	98	0.648
Beck Depression Inventory (BDI 2)	24.29 ± 13.63	23.97 ± 14.43	*t* = 0.114	98	0.910
Hopelessness Scale (H-R-Scale)	69.27 ± 21.84	69.32 ± 22.48	*t* = −0.012	98	0.495
Beck Scale for Suicidal Ideation (BSS) ^a^	9.82 ± 11.47	5.78 ± 8.63	*t* = 1.874	66.09 ^a^	0.065
Suicide Intent Scale (SIS)	12.53 ± 4.50	12.05 ± 4.80	*t* = 0.495	97	00.622
Impulsive Behavior Scale (UPPS)					
Urgency	31.63 ± 6.10	32.21 ± 6.80	*t* = −0.431	97	0.334
Premeditation	22.22 ± 5.82	22.21 ± 5.28	*t* = 0.008	96	0.497
Perseverance	20.51 ± 6.07	21.11 ± 5.45	*t* = −0.507	96	0.307
Sensation-Seeking	29.83 ± 5.93	25.93 ± 7.73	*t* = 2.712	97	0.004 **^b^
German Questionnaire for Assessing Factors of Aggression (K-FAF)					
Spontaneous aggressiveness	13.93 ± 11.05	10.71 ± 7.81	*t* = 1.605	67.01 ^a^	0.057
Reactive aggressiveness	24.90 ± 12.53	23.36 ± 9.79	*t* = 0.692	98	0.245
Excitability	22.98 ± 13.08	19.80 ± 10.96	*t* = 1.317	98	0.095
Self-aggressiveness	28.17 ± 10.64	27.78 ± 10.60	*t* = 0.181	98	0.428
Aggression inhibition	19.32 ± 6.07	20.05 ± 6.14	*t* = −0.591	98	0.278
Sum aggression ^c^	61.80 ± 33.19	53.86 ± 24.29	*t* = 1.308	68.74 ^a^	0.098
State-Trait Anger Expression Inventory-2 (STAXI-2)					
Trait Anger	22.95 ± 7.30	22.10 ± 6.48	*t* = 0.612	98	0.271
Expression Out	14.45 ± 5.57	13.51 ± 4.98	*t* = 0.879	97	0.192
Expression In	22.95 ± 5.46	20.76 ± 5.55	*t* = 1.937	97	0.028 *
Anger Control	29.35± 7.16	29.02 ± 6.72	*t* = 0.236	97	0.407
Childhood Trauma Questionnaire (CTQ)					
Emotional abuse	10.49 ± 5.70	10.86 ± 5.96	*t* = −0.312	96	0.756
Physical abuse	7.60 ± 4.48	7.12 ± 4.03	*t* = 0.558	97	0.578
Sexual abuse	7.56 ± 5.59	6.54 ± 4.21	*t* = 1.027	96	0.307
Emotional neglect	11.58 ± 5.58	12.02 ± 5.13	*t* = −0.406	97	0.686
Physical neglect	8.90 ± 3.67	8.97 ± 3.82	*t* = −0.083	98	0.934

Note. * *p* < 0.05, ** *p* < 0.01. ^a^ adjusted for unequal variances. ^b^ remains significant after a Bonferroni correction. ^c^ includes the subscales spontaneous aggression, reactive aggression and excitability.

## Data Availability

The original contributions presented in the study are included in the article, further inquiries can be directed to the corresponding author/s.
